# Antitheilerial Activity of the Anticancer Histone Deacetylase Inhibitors

**DOI:** 10.3389/fmicb.2021.759817

**Published:** 2021-11-18

**Authors:** Madhumanti Barman, Sonam Kamble, Sonti Roy, Vasundhra Bhandari, Siva Singothu, Debabrata Dandasena, Akash Suresh, Paresh Sharma

**Affiliations:** ^1^National Institute of Animal Biotechnology (NIAB), Hyderabad, India; ^2^National Institute of Pharmaceutical Education and Research (NIPER), Hyderabad, India

**Keywords:** drug repurposing, HDACi, *Theileria annulata*, anticancer, molecular docking

## Abstract

The apicomplexan parasite, *Theileria annulata*, is the most prevalent hemoprotozoan in livestock, causing significant economic losses worldwide. It is essential to develop new and improved therapeutics, as current control measures are compromised by the development of resistance against the only available antitheilerial drug, buparvaquone (BPQ). Histone deacetylase inhibitors (HDACi) were shown to treat cancer effectively and revealed *in vitro* antiparasitic activity against apicomplexan parasites such as *Plasmodium* and *Toxoplasma*. In this study, we investigated the antitheilerial activity of the four anti-cancer HDACi (vorinostat, romidepsin, belinostat, and panobinostat) against the schizont stage of *T. annulata* parasites. All four HDACi showed potent activity and increased hyperacetylation of the histone-4 protein. However, based on the low host cell cytotoxicity and IC_50_ values, vorinostat (0.103 μM) and belinostat (0.069 μM) were the most effective showing antiparasitic activity. The parasite-specific activities of the HDACi (vorinostat and belinostat) were evaluated by western blotting using parasite-specific antibodies and *in silico* analysis. Both vorinostat and belinostat reduced the *Theileria* infected cell viability by downregulating anti-apoptotic proteins and mitochondrial dysfunction, leading to caspase-dependent cell apoptosis. The HDACi caused irreversible and antiproliferative effects on the *Theileria* infected cell lines. Our results collectively showed that vorinostat and belinostat could be used as an alternative therapy for treating *Theileria* parasites.

## Introduction

Bovine Theileriosis (BT), caused by *Theileria* parasites, is an economically significant parasitic disease (Brown, 1990). BT is prevalent in tropical and subtropical countries affecting millions of livestock worldwide (Nene and Morrison, 2016). In India, it is caused by the parasite *Theileria annulata* and *Theileria orientalis* (George et al., 2015a,b), mostly affecting crossbreed animals. In India, BT infections caused by *T. annulata* parasites are life-threatening, leading to the dairy industry’s production and economic loss of $1,295 million/annum (Narladkar, 2018). In the last decade, because of the increase in the number of crossbreed animals, there has been a significant rise in the number of reported cases of *T. annulata* infected animals from India (Kundave et al., 2015; Kumar et al., 2016; Larcombe et al., 2019). The single vaccine and drug buparvaquone (BPQ) are the only hope for fighting against this deadly parasite. The current schizont stage attenuated vaccine (Rakshavac-T) used in India has associated drawbacks like the infrastructure of vaccine production, its distribution, and cold chain maintenance; therefore, it is not commonly used in the field (Jeyabal et al., 2012). This leads to almost complete dependency on chemotherapy for BT treatment. In countries like Tunisia, Iran, and Sudan, BPQ resistance is reported from the field. However, in the published studies, the level of resistance or the prevalence of BPQ resistant *Theileria* parasites have not been done (Mhadhbi et al., 2010, 2015; Sharifiyazdi et al., 2012; Chatanga et al., 2019). Hence, there is an urgent need to discover new antitheilerial drugs/compounds to control the disease.

As new drug discovery takes a long time, drug repurposing is one approach that has helped researchers discover the unknown potential of the clinically approved drugs (Ashburn and Thor, 2004; Nwaka and Hudson, 2006). Identifying drugs that inhibit the parasite genes involved in transcriptional regulation, posttranslational modifications, or epigenetic regulation seems a good strategy for searching for new antiparasitic drugs (Andrews et al., 2014). In eukaryotes, HDACs have been shown to regulate multiple essential pathways, and abnormal alterations in these enzymes can lead to apoptosis or cancerous growth in cells (Gallinari et al., 2007; Li and Seto, 2016). HDAC inhibitors (HDACi) like vorinostat, romidepsin, belinostat, and panobinostat are FDA approved to treat different cancers (Grant et al., 2007; Prince and Dickinson, 2012; Thompson, 2014; Garnock-Jones, 2015). The *in vitro* antiparasitic activity of these four inhibitors has been previously investigated in protozoa parasites like *Plasmodium*, *Trypanosoma*, *Leishmania*, and *Schistosoma* (Engel et al., 2015; Chua et al., 2017). In *P. falciparum* and *P. knowlesi* parasites, all four HDACi have shown potent antiplasmodial activity. Because of differences between the human and parasitic HDACs, these enzymes seem promising targets for developing new generation antitheilerial drugs.

Keeping in mind the unavailability of backup drugs for treating BT infections, we planned to test known drugs to find their ability to target unique or unexplored pathways specific to the parasite. In this study, we tested antitheilerial activity of the four HDACi: vorinostat, romidepsin, belinostat, and panobinostat against the *T. annulata* parasites. These HDACi have never been targeted before for their antitheilerial activity and can be a new addition as an alternative therapy against *T. annulata* parasites. We have also investigated the hyperacetylation profiles of the *Theileria* infected cells after treatment with the compounds. Additionally, we have done molecular docking studies for showing the binding of HDACi to the *Theileria* specific proteins using *in silico* studies.

## Materials and Methods

### Compounds

Buparvaquone (Cat No. B4725), belinostat (Cat no. A4096), and panobinostat (Cat no. 13280) were purchased from Apex Bio. Vorinostat (SAHA) was purchased from EpiGentek (M41000-2), and romidepsin (17130) was purchased from Cayman. All HDACi were prepared as 10–20 mM stock solutions in phosphate-buffered saline (PBS). BPQ was prepared as a 10 mM stock solution in 100% DMSO.

### *Theileria annulata* Growth Inhibition Assays

*Theileria annulata* infected bovine cells were derived previously from the clinically infected cattle and cultured in the RPMI 1640 medium (Sigma Aldrich) supplemented with 10% heat-inactivated fetal bovine serum and 100 μg/mL Penicillin-Streptomycin at 37°C in a CO_2_ incubator (George et al., 2015b; Roy et al., 2019). Antitheilerial activity of the compounds was analyzed by incubating different concentrations of compounds to *T. annulata* infected cells. Briefly, 5 × 10^3^ *T. annulata* infected cells were seeded per well in 96 well plates in 200 μL medium at 37°C for 4 h. All four HDACi were serially diluted and added to the cells in the 96 well plate for 48 h. After 48 h, 30 μL resazurin dye (1.5 mM) was added to each well, and the fluorescence intensity of the cells was measured at 570 nm for accessing the viability of the cells based on the previously published method (Kulshrestha et al., 2013). BPQ was used as a positive control in all the assays. Each experiment was performed at least thrice independently in triplicates. The cytotoxicity profiles of the compounds were evaluated in BOMAC (Bovine macrophage cell Line) cell line using the standard protocol.

### Protein Hyperacetylation Assay

Hyperacetylation assays were carried out using the protein lysate of the *T. annulata* infected bovine cells. Briefly, 1 × 10^5^ cells were incubated for 3 h with IC_50_ concentration of test compounds (1X and 5X), and untreated cells were included as a control. BPQ treated cells were used as a negative control. *T. annulata* infected cells were then pelleted and washed thrice with 1X PBS before resuspending the cells for lysis in RIPA (Radio-Immunoprecipitation Assay) buffer. After sonication and centrifugation of the lysed cells, proteins were quantified using the BCA protein assay kit. SDS-PAGE loading dye was added to the sample, followed by denaturation (97^°^C, 5 min) and separation on SDS-PAGE. Proteins were then transferred to polyvinylidene difluoride (PVDF) membrane, and western blotting was done using anti-tetra acetyl histone H4 antibody (1:2,000, Sigma-Aldrich, 05-1355) and goat anti-mouse IgG secondary antibody (1:2,000) using chemiluminescent reagent (Takara). Histone H3 (1:2,000, CST, 9715S) was taken as the loading control. Membranes were imaged using the Biorad ChemiDoc Imaging system. Western blot images were processed in Image J software for protein quantification using the relative density method. Band intensities of the H3 (loading control) and anti-tetra acetyl histone H4 [the protein of interest (POI)] were quantified by taking the area of interest. Intensities were normalized by dividing the respective value with one of the samples for loading control and POI. Relative expression was calculated by dividing the normalized intensity of POI by its respective loading control.

### Immunofluorescence Assay

5 × 10^4^ *T. annulata* infected cells were incubated with IC_50_ concentration of HDAC compounds (vorinostat, romidepsin, and belinostat) with untreated cells as control. BPQ treated cells were used as the negative control. Cells were pelleted down after 3 h of incubation and washed thrice with 1X PBS. Next, the cells were fixed using 4% paraformaldehyde (37^°^C, 10 min) followed by 1X PBS washing and permeabilization by 0.1% Triton X-100. Permeabilized cells were incubated for 1 h with blocking buffer (2% BSA in 1X PBS) at room temperature. Cells were then incubated with anti-acetyl histone H4 (1:250, Sigma-Aldrich, 05-1355) antibody overnight at 4^°^C. The primary antibody was then discarded, and the slide was washed three times in PBS, followed by incubation with goat anti-mouse Cruz Fluor 555 secondary antibody (1:250, Santacruz) for 1 h at room temperature. Cells were further washed with 1X PBS, and gold antifade mountant with DAPI (1 μg/mL) was used to stain the nucleus. Images were recorded in the Airyscan microscope (Zeiss), and ZEN Blue software was used for analysis.

### Western Blotting and Mitochondrial Membrane Potential

For western blot analysis, total proteins from the *T. annulata* infected cells were fractioned on 8% polyacrylamide gels before and after 48 h treatment of vorinostat and belinostat compounds. For checking the parasite-specific effect of these compounds, blotting was done for detection of *TaSP* (*Theileria annulata* surface protein) using rabbit anti-*TaSP* peptide antibody (1:3,000) and mouse anti-β-actin (1:1,000) as a loading control. The primary antibody was then discarded, and the membrane was washed thrice in PBS, followed by incubation with horseradish peroxidase-conjugated IgG secondary antibody (1:1,000; Thermo Fisher Scientific) for 1 h at room temperature. The membrane was imaged using the chemiluminescent reagent (Takara) on the Biorad ChemiDoc Imaging system.

For mitochondrial membrane potential analysis, *T. annulata* infected cells treated with and without belinostat and vorinostat drugs were incubated with a JC-1 probe. BPQ treated cells were used as a control in the experiment. After 48 h of drug treatment, cells were incubated with 2.5 μL of JC-1 dye for 20 min in the dark at 37^°^C. After washing, cells were resuspended in 500 μL of cell staining buffer. Data acquisition was made on the BD LSR Fortessa, followed by analysis using the Flow Jo software (Tree Star Inc., Ashland, OR). Mitochondrial depolarization was quantified by taking the ratio of red to green fluorescence emission intensity. All the fluorescence assays were carried out in two independent experiments.

### Analysis of Cell Death Using Flow Cytometry

Annexin V-FITC and propidium iodide (PI) staining was done to investigate the cell death mechanism using flow cytometry. Briefly, 1 × 10^5^ cells/well were incubated with 1X IC_50_ of test compounds (vorinostat and belinostat) with or without z-VAD-fmk (2 μM) for 48 h. Staurosporine (1 μM) with or without z-VAD-fmk (2 μM) was taken as the positive control. After 48 h, cells were washed with PBS and incubated with annexin V binding buffer (500 μL/tube) containing 5 μL annexin V and 10 μL PI for 15 min at 37^°^C. Data acquisition was made on the BD LSR Fortessa, followed by analysis using the Flow Jo software (Tree Star Inc., Ashland, OR) for detecting the % of apoptosis or necrosis in cells. Assays were performed in duplicate in three independent experiments.

### Reverse Transcriptase-Polymerase Chain Reaction

HDACi (vorinostat and belinostat) treated and untreated cells were collected, total RNA was extracted using Trizol reagent, and 5^°^μg of total RNA was reverse transcribed for cDNA synthesis using a Primescript cDNA synthesis Kit (Takara) according to the manufacturer’s protocol (Dandasena et al., 2018). The mRNA expression of matrix metalloproteinase 9 (MMP9) and B-cell lymphoma 2 (Bcl-2) gene was detected by real-time PCR using a BioRad CFX96 Touch System (Biorad). Relative target gene expression was calculated using the 2^–ΔΔCT^ method. Primer sequences used are as follows: MMP9: Forward-5′ CCCATTAGCACGCACGACAT-3′, Reverse 5′-TCACGTAGCCCACATAGTCCA-3′; HRPT1: Forward-5′-TGTGGCCAGCTTAATAG-3′, Reverse5′- GGCTCGTAGTGCA AATGAAG-3′, Bcl-2: Forward-5′- GATGACCGAGTACCT GAACC -3′, Reverse 5′- AGCCAGGAGAAATCAAACAGG-3′.

### Homology Modeling and Molecular Docking

Since no crystal structure is available for the *TaHDAC1* putative protein, we used its amino acid sequence (TA12690) for searching its homologous proteins with available crystal structure in the Protein Data Bank (PDB). Human HDAC2 (PDB accession No. 5IWGA) was found to be a suitable template for modeling with 62.91% similarity to the TaHDAC1 protein at a resolution of 1.66 Å (Figure 4). Homology modeling of putative histone deacetylase of *T. annulata* (TA12690) was carried out using SWISS-MODEL Homology Modeling server.^1^ Ramachandran plot, QMEAN score plot, and Local quality estimates assessed the quality of the modeled protein. The Ramachandran plot was generated using the PROCHECK program in Structure Analysis and Verification Server (SAVES) (Laskowski et al., 1996). The protein’s ligand-binding site was determined using the 3DLigandSite prediction server and the previously published literature (Marks and Breslow, 2007; Wass et al., 2010). The modeled protein was saved in PDB format and docked using Schrodinger Maestro (version 12.2).

The grid generation module did the catalytic binding site’s visualization and characterization. The ligands structure file was downloaded from the PubChem database [vorinostat (ID-5311), panobinostat (ID- 6918837), belinostat (ID-6918638), romidepsin (ID-5352062)] and prepared for docking to the modeled protein. The ligands were optimized using the OPLS3e force field in the Ligprep module, followed by docking into the generated receptor grid using the sitemap option in Schrodinger Maestro. The ligand conformation having the lowest binding energy was considered for all the inhibitors.

### Reversibility of Growth Inhibition After Treatment With Histone Deacetylase Inhibitor

*Theileria* infected cells were treated with IC_50_ concentration of vorinostat and belinostat compounds for 48 h to check the effect on parasite growth. After 48 h, drug pressure was removed, and parasites were grown in a traditional medium without the HDACi. The proliferation of the *T. annulata* cells was monitored by trypan blue assay for the next 12 days for assessing the effect of drug treatment.

## Results

### Histone Deacetylase Inhibitors Showed Antitheilerial Activity Against *Theileria annulata* Parasites

**FIGURE 1:**
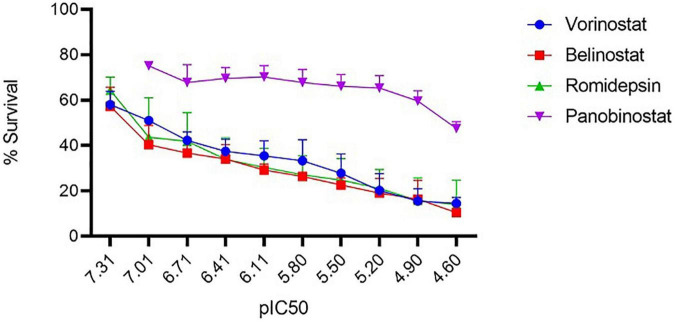
*In vitro* antitheilerial activity of histone deacetylase (HDAC) inhibitors. Dose-response curves against the four HDACi in *T. annulata* infected cells. IC_50_ was calculated using a resazurin dye-based assay. The IC_50_ values of vorinostat, belinostat, romidepsin, and panobinostat was 0.103 (±0.005) μM, 0.069 (±0.004) μM, 0.20 (±0.006) μM, and 20.80 (±3.11) μM, respectively. IC_50_ values are represented as the negative log of IC_50_ in Molar referred to as pIC_50_ (ranged from 7.31 to 4.60 referring to IC_50_ concentration from 0.097 to 25 μM). The graph represents the mean % survival at different concentrations of HDACi. ± represents the standard deviation (SD) from the three independent experiments. All the experiments were done in triplicates.

For assessing the antitheilerial activity of the HDACi (vorinostat, belinostat, romidepsin, and panobinostat), *in-vitro* cultured *T. annulata* parasites were challenged with different concentrations of the compounds. BPQ was included as a control in the study. Except for panobinostat, all the other inhibitors showed potent antiparasitic activity based on the observed IC_50_ values (Figure 1). The values of vorinostat, belinostat, romidepsin (<0.3 μM) were at least 20 times lower than that of the panobinostat (20 μM) compound (Table 1). These four HDACi were previously reported to be effective (IC_50_ ≤ 0.1 μM) against *P. falciparum* and *P. knowlesi* strains (Engel et al., 2015). A comparison was made for the effectiveness of the HDACi based on the IC_50_ values between the *Theileria* and *Plasmodium* parasites. The vorinostat, belinostat, and romidepsin values in *T. annulata* were similar to previously published data in *Plasmodium* parasites (IC_50_ ≤ 0.2 μM) (Engel et al., 2015). However, panobinostat behaved differently, showing antiparasitic activity at significantly higher drug concentrations in *T. annulata* cells compared to *Plasmodium* parasites (IC_50_ ≤ 0.03 μM) (Engel et al., 2015).

**TABLE 1:** *In vitro* antitheilerial activity of histone deacetylase (HDAC) inhibitors against *T. annulata* infected cells.

Compound	Structure	PubChem CID	*T. annulata* IC50 (μM)	Mammalian cell IC50 (μM)	SI
Vorinostat		5311	0.103 (±0.005)	>25	>140
	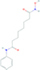				
Belinostat		6918638	0.069 (±0.004)	9.875 (±3.712)	195
	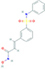				
Romidepsin		5352062	0.200 (±0.006)	0.296 (±0.029)	1.451
	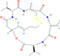				
Panobinostat		6918837	20.800 (±3.110)	nd	nd
	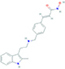				
Buparvaquone		71768	0.153(±0.011)	>1.500	10.239
	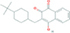				

*Nd, not determined. SI, (Mammalian cells IC_50_)/(T. annulata parasite IC_50_); larger values = greater parasite selectivity.*

### *In vitro* Cytotoxicity of Histone Deacetylase Inhibitors

Since only three HDACi (vorinostat, belinostat, and romidepsin) had potent *in-vitro* activity (IC_50_ ≤ 0.3 μM), we decided to focus on these compounds for further studies. The *in vitro* cytotoxicity was assessed for the three HDACi against the BOMAC cells using a resazurin dye-based assay. Vorinostat and belinostat were non-toxic based on the IC_50_ values of the assay (Table 1). In contrast, romidepsin was equally toxic (>0.2 μM) on mammalian cells compared to *T. annulata* infected cells. The SI values of vorinostat and belinostat in *T. annulata* compared to mammalian cells (SI 140 and 195, respectively; Table 1) were higher than previously published data for *Plasmodium* parasites (SI 140 and 195, respectively) indicating greater selectivity for *Theileria* parasites. Our results with romidepsin were in sync with the previously published cytotoxicity results in *Plasmodium* species (Engel et al., 2015).

### Histone Deacetylase Inhibitors Leads to Hyperacetylation of *Theileria annulata* Infected Cells

**FIGURE 2:**
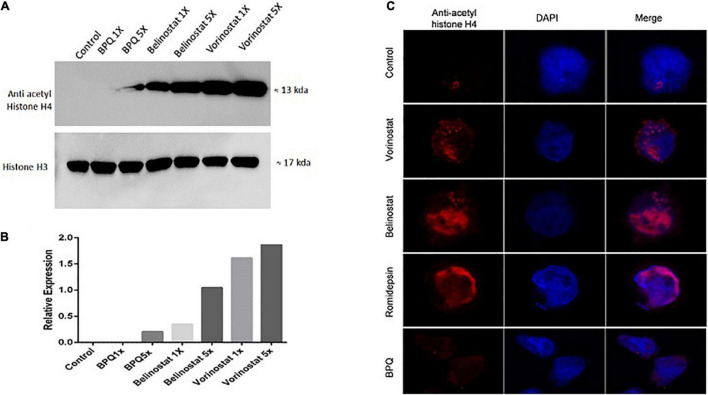
Acetylation profiles of *T. annulata* infected cells after 3 h of treatment with histone deacetylase (HDAC) inhibitors. **(A)** Western blot analysis of the *T. annulata* infected cells treated with 1× IC_50_ and 5× IC_50_ concentrations of different HDACi (vorinostat, belinostat, and romidepsin) and controls (without treatment and with BPQ treatment) using mouse anti- acetyl histone H4 antibody. Histone H3 was taken as the loading control. **(B)** The graph shows the relative quantification of the H4 as detected in the western blot. **(C)** Immunofluorescence staining of *T. annulata* infected cells with 1× IC_50_ HDACi (vorinostat, belinostat, and romidepsin) after 3 h of treatment. BPQ was taken as control.

For checking hyperacetylation of proteins in *Theileria* infected cells, cell lysate was prepared after 3 h of treatment with HDACi (vorinostat, belinostat, and romidepsin). For quantitative assessment, *T. annulata* infected cells were treated with 1X and 5X concentrations of the IC_50_ values of the inhibitors. Vorinostat, belinostat, and romidepsin treatment clearly showed increased acetylation of H4-proteins (∼13–17 KDa) compared to untreated control and BPQ treated cells using pan acetyl histone antibody in western blot (Figures 2A,B). We next checked the hyperacetylation using IFA with the same antibody with cells treated with 1X concentration (IC_50_) of the three inhibitors. The fluorescence microscopy images confirmed the increased hyperacetylation in the *T. annulata* infected cells compared to control, and BPQ treated cells (Figure 2C).

We also investigated whether the increase in hyperacetylation due to HDACi treatment affects the virulence of the parasite. MMP9 gene expression was analyzed in the HDACi treated and untreated samples to quantify the effect on virulence. Decreased expression of host MMP9 gene has been previously linked to attenuation or decrease in the virulence of the *T. annulata* parasites (Echebli et al., 2014). Bcl-2 gene, a well-known marker for apoptosis, was also included in the study. The quantitative SYBR green-based PCR analysis showed a twofold increase in the MMP9 gene expression after treatment with belinostat. However, no differential expression was found in the MMP9 gene after treatment with vorinostat. Anti-apoptotic gene Bcl-2 was found to be downregulated in both the vorinostat and belinostat treated samples (Supplementary Figure 1).

### Histone Deacetylase Inhibitors Kills the Parasite Explicitly in an Irreversible Manner and Damages the Mitochondrial Potential of *Theileria annulata* Infected Cells

**FIGURE 3:**
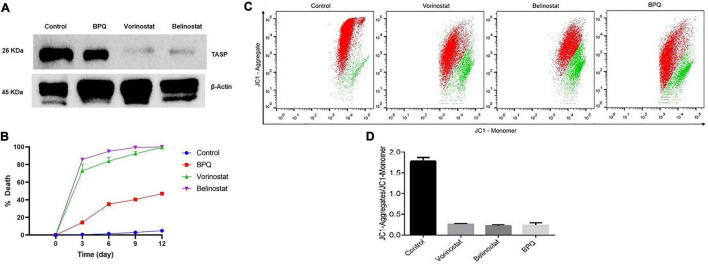
Effect of Belinostat and Vorinostat on the parasite and its mitochondrial membrane potential: **(A)** Western blot analysis of the *T. annulata* infected cells treated with IC_50_ concentrations of vorinostat and belinostat (after 48 h) and controls [without drug treatment and buparvaquone (BPQ) treatment] using mouse anti-*TaSP* antibody and mouse anti-β-actin as a loading control. **(B)** Reversibility of growth inhibition after treatment with histone deacetylase (HDAC) inhibitor. After 48 h, HDACi and BPQ drug pressure were removed, and parasite growth was monitored for 12 days in the traditional medium. The experiment was repeated thrice. **(C)** Flow cytometry analysis using JC1 dye to analyze vorinostat and belinostat effect on the mitochondrial membrane potential of *T. annulata* infected cells. Mitochondria depolarization was quantified by taking the ratio of red to green fluorescence emission intensity. The JC1-aggregate and the JC1-monomer are represented by red and green color, respectively, in the dot-plot. **(D)** The graph shows the ratio of JC1-aggregate and the JC1-monomer from the flow cytometer analysis.

*Theileria annulata* infected cells were incubated with belinostat (0.069 μM) and vorinostat (0.103 μM) for 48 h. After treatment, the cells were labeled with anti-*TaSP* (parasite-specific) and anti-β-actin (host-specific) antibodies, followed by western blotting. There was a significant decrease in the *TaSP* protein levels after 48 h treatment with both the compounds (Figure 3A). However, the intensity of the β-actin band was similar in the treated and untreated samples. BPQ treated samples used as a positive control also showed a decrease in the band intensity of the *TaSP* protein.

We also investigated whether the antiparasitic effect of HDACi is reversible after the removal of the drug pressure. Treatment of *T. annulata* infected cells with belinostat and vorinostat for 48 h resulted in the complete and irreversible suppression of the parasite growth even after drug pressure withdrawal (Figure 3B). There was no recovery till 12 days after drug withdrawal of the parasite.

The mitochondrial membrane potential of the *T. annulata* infected cells treated with HDACi (belinostat and vorinostat) was measured using JC1 dye to analyze their effect on the mitochondrial function. The membrane potential was measured by calculating the mean red fluorescence intensity (JC1-Aggregate) to mean green fluorescence intensity (JC1-Monomer). The flow cytometer-based analysis identified a significant decrease in the ratio of red to green fluorescence intensity in the treated cells as compared to the untreated cells (Figures 3C,D).

### Histone Deacetylase Inhibitors Induces Caspase-Dependent Apoptosis in the *Theileria* Infected Cells

**FIGURE 4:**
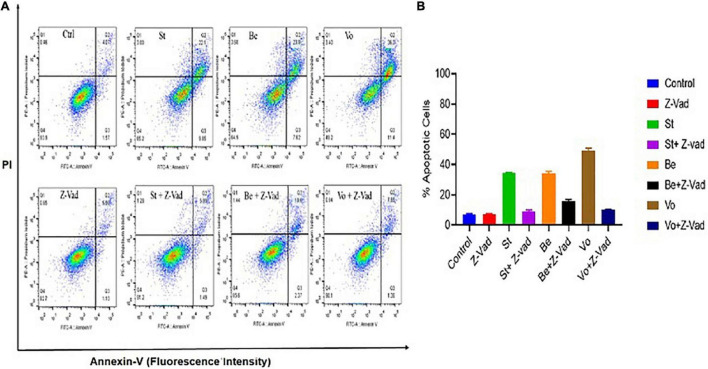
Histone deacetylase inhibitors (HDACi) treatment induces caspase-dependent apoptosis in the *Theileria* infected cells. **(A)** The percentage of apoptosis was quantified by flow cytometry analysis using annexin V and PI staining. **(B)** Quantitative analysis of the apoptotic cells based on an average of three independent experiments (mean ± SD).

Flow cytometry analysis was performed to analyze whether belinostat and vorinostat-induced cell death is associated with apoptosis. Staurosporine (apoptosis-inducing agent) and z-VAD-fmk (pan-caspase inhibitor) were used as a control to examine the caspase-dependent apoptosis in HDACi treated *Theileria* infected cells (Belmokhtar et al., 2001). After 48 h of treatment, belinostat and vorinostat significantly promote caspase-dependent apoptosis in the infected cells (Figures 4A,B). Belinostat and vorinostat-induced apoptosis was completely blocked by the broad caspase inhibitor z-VAD-fmk, demonstrating that caspases were involved in the death process (Figures 4A,B). Figure 4A shows that belinostat and vorinostat significantly promote cell apoptosis, the percentages of apoptotic cells were as follows: control: 6.88 ± 0.63%, z-VAD-fmk: 7.37 ± 0.20%, staurosporine: 34.59 ± 0.13%, staurosporine + z-VAD-fmk: 9.07 ± 0.71%, belinostat: 34.26 ± 0.97, vorinostat: 49.37 ± 1.18%, belinostat + z-VAD-fmk: 15.66 ± 0.99%, vorinostat + z-VAD-fmk: 10.21 ± 0.19%.

### *In silico* Studies Predict TaHDAC1 to Be the Target of Histone Deacetylase Inhibitors

**FIGURE 5:**
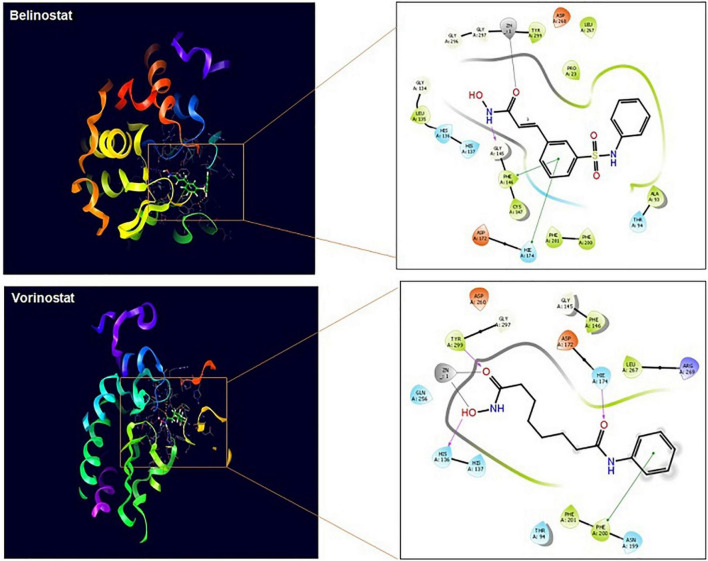
TaHDAC1 homology model structure with docked ligands. The first figure shows the secondary structure representation of the homology model of TaHDAC1. The second part of the figure shows the 2D interaction between the inhibitor and the modeled protein amino acid residue. The docking poses are shown for ligands (vorinostat) and (belinostat) in *TaHDAC1*. Critical interactions with zinc atom (gray line), π−π interactions (green line), and hydrogen bonds (pink line) are shown.

Vorinostat and belinostat hinder HDAC enzyme activity leading to hyperacetylation of proteins and parasite death in *P. falciparum* and *P. knowlesi* parasites (Sumanadasa et al., 2012; Chua et al., 2017). In *Plasmodium*, five different HDAC enzymes are reported, which can have a role in the acetylation and deacetylation of histones. We found homologs of all the five plasmodial genes in *T. annulata* genome (Supplementary Table 1). The antiplasmodial activity of the four HDACi used in this study was previously linked to inhibition of *PfHDAC1* (PlasmoDB—gene ID PF3D7_0925700) and PkHDAC1 gene in *P. falciparum* and *P. knowlesi*, respectively (Engel et al., 2015). We searched for the homolog of the *PfHDAC1* and *PkHDAC1* genes in the *T. annulata* database (PiroplasmaDB). The homology analysis identified the *Ta12690* gene (*TaHDAC1*, putative) as the *Plasmodium* species closest match. As the crystal structure of both *Plasmodium* and *Theileria* HDAC is not available, we used TaHDAC1 as a template and found human HDAC2 (PDB No. 5IWGA) to be very similar to the *Theileria* protein (Supplementary Figure 2). Using the crystal structure of the human HDAC2, we draw a three-dimensional homology structural model of *TaHDAC1* to examine the predicted binding mode of these ligands in the *Theileria*. The model’s quality assessment was done based on the QMEAN score (–0.89) and GMQE (0.70) values; our structure was found to be within the allowed limits of modeling. The local quality estimates for the 3D model showed two regions with a score below 0.6, but the ligand binding/active site have scored above 0.6. The Ramachandran plot showed 91.4 and 8.6% of residues from the model located in the most favored or allowed regions (Supplementary Figure 3). We next docked the ligands to find the possible binding sites in the TaHDAC1. Based on the 3DLigandSite prediction tool, the expected binding of ligands was near the residues His136, His137, Asp172, Val173, His174, Asp260, Gly296, Gly297, Gly298, and Try299 of TaHDAC1. The docking of TaHDAC1 revealed hydroxamate binding of the ligands (vorinostat and belinostat) to the zinc ion in the active site (Figure 5). The vorinostat and belinostat made hydrogen bonds (His136, His174, Try299, and Gly145) and pi-pi (Phe200 and His174, Phe146) interactions near the active site residues in *TaHDAC1* (Figure 5). The docked ligands showed a high docking score of –5.233 and –8.202, respectively. Since panobinostat was previously reported to be the most potent inhibitor of the *P. falciparum* and *P. knowlesi* parasites, we compared differences in its binding to *Plasmodium* and *Theileria* HDAC1. Although panobinostat showed binding to zinc ion in the catalytic site, there was no interaction with the active site residues in *TaHDAC1* (data not shown). Thus binding of hydroxamic acid-based compounds (vorinostat and belinostat) in the active site pocket might inhibit *TaHDAC1* activity resulting in hyperacetylation of the proteins and ultimately parasite death.

## Discussion

*Theileria annulata* is the most common hemoprotozoan parasite infection in livestock, causing high mortality and production losses. The disease control efforts are badly affected due to BPQ resistance, the only available drug used for treating the parasites (Mhadhbi et al., 2010, 2015; Sharifiyazdi et al., 2012; Chatanga et al., 2019). It is essential to find new therapeutic options by identifying new targets or by repurposing drugs for combatting the deadly parasite. Drug repurposing has emerged as a very effective tool to bypass the traditional method of drug discovery. Some of the common repurposed drugs include thalidomide and metformin for cancer and antibacterials such as azithromycin, tetracyclines, sulfonamides, and clindamycin for parasitic diseases (Nzila et al., 2011; Zhang et al., 2020). This study utilized the repurposing strategy by targeting epigenetic regulatory enzymes to find new treatment options against *T. annulata* parasites.

Four HDACi (vorinostat, belinostat, panobinostat, and romidepsin) that have been clinically approved for treating various cancer forms were evaluated for their antitheilerial activity. These HDACi are also well-studied in other protozoan parasites like *Plasmodium, Trypanosoma, Leishmania*, and *Schizostoma*. In *Plasmodium*, these compounds are shown to be a potential target for the treatment of *P. falciparum*, *P. knowlesi*, *P. berghei*, and *P. vivax* parasites (Andrews et al., 2008; Agbor-Enoh et al., 2009; Chaal et al., 2010; Marfurt et al., 2011; Chua et al., 2017). Vorinostat, belinostat, and panobinostat are hydroxamic acids like compounds shown to inhibit the pan-HDACs, while romidepsin is a cyclic peptide inhibiting class 1 HDACs (Mottamal et al., 2015). With an IC_50_ of <0.3 μM, belinostat, vorinostat, and romidepsin showed potent activity against the clinical isolates of the *T. annulata* parasites. Although effective in killing the parasites, the panobinostat had a significantly higher IC_50_ (20 μM) than other HDACi. Romidepsin showed high host cell cytotoxicity, which was in line with other previously reported studies where despite its effectiveness in killing *Plasmodium* and *Trypanosoma* parasites, it was not considered as a promising target (Engel et al., 2015). In previous reports, belinostat, vorinostat, and panobinostat have been shown to have potent and selective activity against the *Plasmodium* parasites, with panobinostat being the most effective (Engel et al., 2015). It was surprising that panobinostat was the least effective against the *Theileria* parasites, which might be due to changes in the gene sequence or the different life cycles of the two parasites. Vorinostat has also been recently reported to be equally effective in killing *Toxoplasma gondi* parasites (Araujo-Silva et al., 2021). The compounds (belinostat and vorinostat) completely and irreversibly halted *T. annulata* proliferation even after removing the drug pressure.

Furthermore, we also revealed that treatment with belinostat and vorinostat downregulates anti-apoptotic proteins and mitochondrial dysfunction, leading to cell apoptosis. Our flow cytometry data based on the annexin V and PI labeling showed that belinostat and vorinostat inhibit the growth of the *Theileria* infected cells mainly by inducing apoptosis while necrosis was observed in a minimal number of cells. We also confirmed that apoptosis induced by the two HDACi was completely blocked by incubation with the caspase inhibitor, z-VAD-fmk, suggesting caspase-dependent cell death. Belinostat and vorinostat have previously been reported to induce similar cell death mechanisms in different cancer cells (Petruccelli et al., 2011; Sarfstein et al., 2011; Ong et al., 2016; Tuncer, 2021). Our data indicate belinostat and vorinostat to be promising leads for developing future parasite selective therapy based on the low host cell cytotoxicity and potent antiparasitic activity.

The HDACi are known to regulate gene expression by hyperacetylation of the histone proteins (H3 and H4), which is used as a marker in *P. falciparum* for confirming their parasite-specific inhibitory activity (Darkin-Rattray et al., 1996; Andrews et al., 2008; Chaal et al., 2010; Chua et al., 2017). Since *Plasmodium* and *Theileria* are apicomplexan parasites, we next checked for the hyperacetylation profiles of histone-4 protein after exposure to compounds (belinostat and vorinostat) in *T. annulata* infected cells. Hyperacetylation was observed in *T. annulata* infected cells treated with belinostat and vorinostat compared to untreated cell lines. The hyperacetylation profiles were similar to the previous studies in *Plasmodium* (Chua et al., 2017). As belinostat and vorinostat are not cytotoxic to host cells, these drugs may inhibit parasite HDACs, similar to what is shown for the other apicomplexan parasites. We confirmed this parasite-specific effect after treating these two compounds by quantifying parasite-specific protein (*TaSP*) using western blotting. The activity of the *Plasmodium* HDAC1 enzyme is previously shown to be inhibited by these inhibitors. In the absence of the recombinant HDAC1 of *T. annulata*, we did *in silico* studies to identify whether these HDACi target parasite-specific enzymes. The docking studies confirmed the binding of belinostat and vorinostat in the active site of the TaHDAC1 enzyme, which is in line with what was reported for the PfHDAC1 and PkHDAC1 (Engel et al., 2015; Chua et al., 2017).

In summary, this is the first study showing the antiparasitic activity and mechanism of action of HDACi in the *T. annulata* parasites. Our data clearly shows that drugs belinostat and vorinostat have potent activity against the *Theileria* infected cells. In the future, we will also like to check the activity and pharmacokinetics of these compounds in the *in vivo* experiments. We also plan to make parasite-specific analogs of these inhibitors, which can develop alternative therapies for treating *Theileria* parasites.

## Data Availability Statement

The original contributions presented in the study are included in the article/Supplementary Material, further inquiries can be directed to the corresponding author.

## Author Contributions

PS designed the experiments and wrote the manuscript. MB, SK, SR, VB, SS, DD, and AS did the experiments and analysis. MB, SK, SR, VB, SS, DD, and AS helped in designing the study, data analysis, and manuscript editing. VB edited the manuscript. All authors gave approval to the final version of the manuscript.

## Conflict of Interest

The authors declare that the research was conducted in the absence of any commercial or financial relationships that could be construed as a potential conflict of interest.

## Publisher’s Note

All claims expressed in this article are solely those of the authors and do not necessarily represent those of their affiliated organizations, or those of the publisher, the editors and the reviewers. Any product that may be evaluated in this article, or claim that may be made by its manufacturer, is not guaranteed or endorsed by the publisher.

## Footnotes

1
https://swissmodel.expasy.org/

## Supplementary Material

Supplementary Figure 1 qRT-PCR determination of MMP9 and Bcl-2 gene expression relative to HDACi treatment in *Theileria* infected cells. Gene expression levels were normalized to an internal control HPRT and fold change was calculated with respect to expression levels in untreated cells.

Supplementary Figure 2 Alignment of the Putative *T. annulata* HDAC1 to the Human HDAC 2(Uniprot—Q92769, PDB structure—5IWG).

Supplementary Figure 3 Ramachandran plot of *T. annulata* HDAC1 homology model.
