# Supplementary material for: Antitheilerial Activity of the Anticancer Histone Deacetylase Inhibitors
Source: Front Microbiol. 2021 Nov 18;12:759817. doi: 10.3389/fmicb.2021.759817 (PMC8640587; doi:10.3389/fmicb.2021.759817)
Supplement: Supplementary file 1 [file Table_1.docx]

Table S1 - HDAC amino acid sequence identity (%) for *P. falciparum*& *T.annulata*

| **Gene Name** | ***P. falciparum* gene ID** | ***Theileria annulata* gene ID** | **% Identity** |
| --- | --- | --- | --- |
| **HDAC1** | PF3D7_0925700 | TA12690, TA18230, TA17590 | 73, 33, 24 |
| **HDAC2** | PF3D7_1472200 | TA17590, TA18230, | 44, 30 |
| **HDAC3** | PF3D7_1008000 | TA18230 | 44 |
| **Sir2A** | PF3D7_1328800 | TA20415 | 33 |
| **Sir2B** | PF3D7_1451400 | TA20415 | 33 |
