# Supplementary figures and images for: Antitheilerial Activity of the Anticancer Histone Deacetylase Inhibitors
Source: Front Microbiol. 2021 Nov 18;12:759817. doi: 10.3389/fmicb.2021.759817 (PMC8640587; doi:10.3389/fmicb.2021.759817)

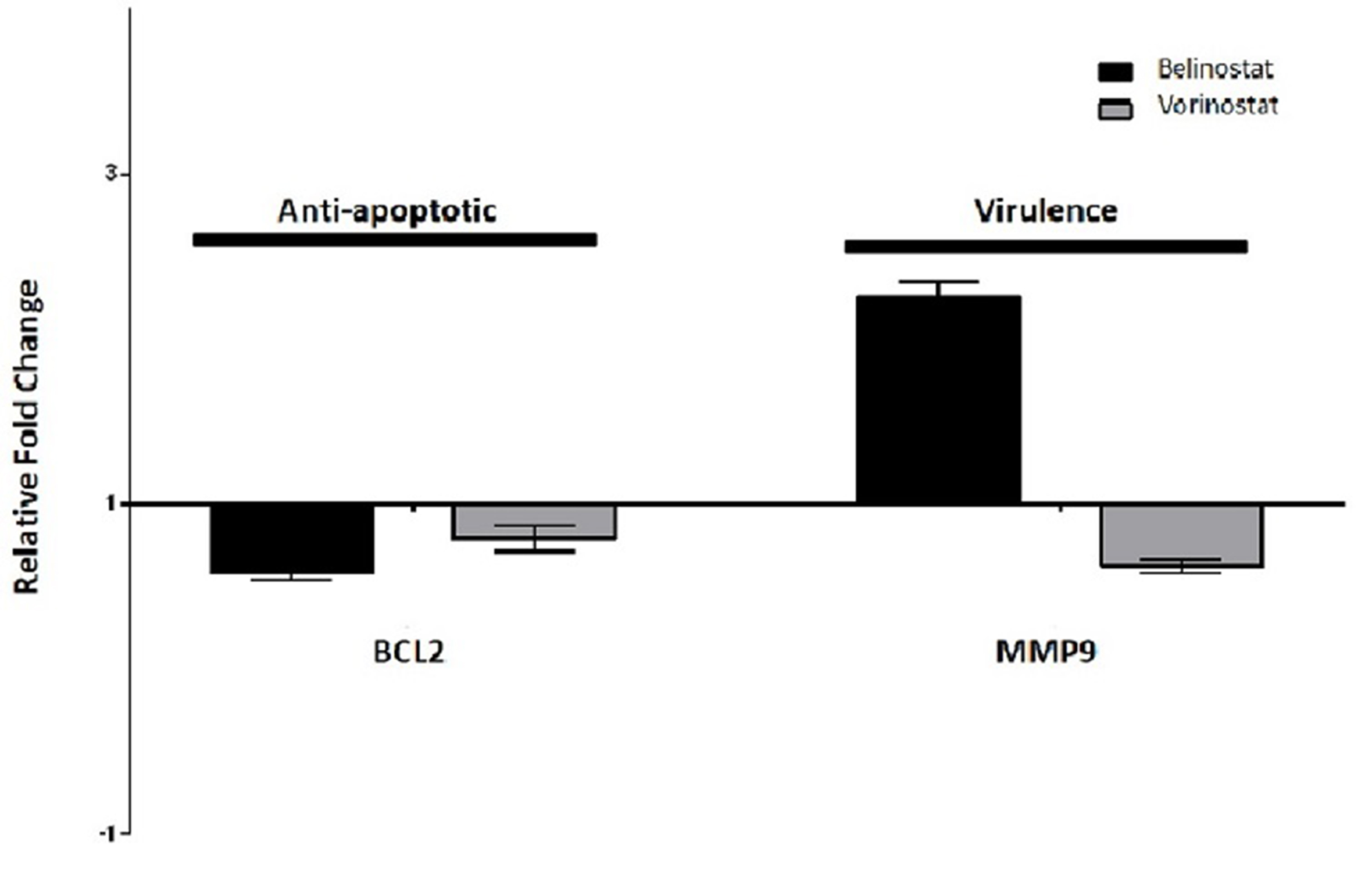

Supplement: Supplementary Figure 1 — qRT-PCR determination of MMP9 and Bcl-2 gene expression relative to HDACi treatment in Theileria infected cells. Gene expression levels were normalized to an internal control HPRT and fold change was calculated with respect to expression levels in untreated cells. [file Image_1.JPEG]

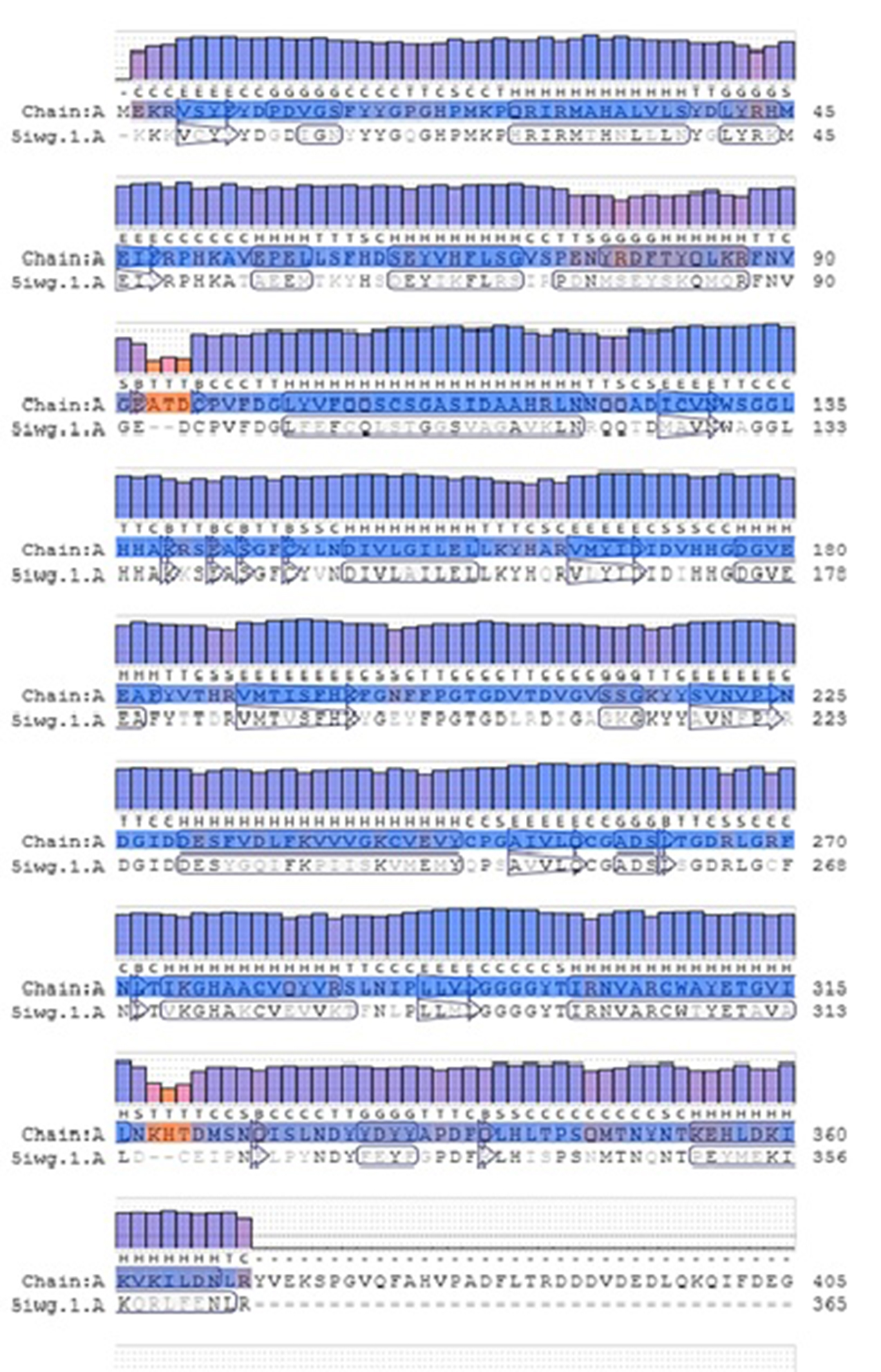

Supplement: Supplementary Figure 2 — Alignment of the Putative T. annulata HDAC1 to the Human HDAC 2(Uniprot—Q92769, PDB structure—5IWG). [file Image_2.JPEG]

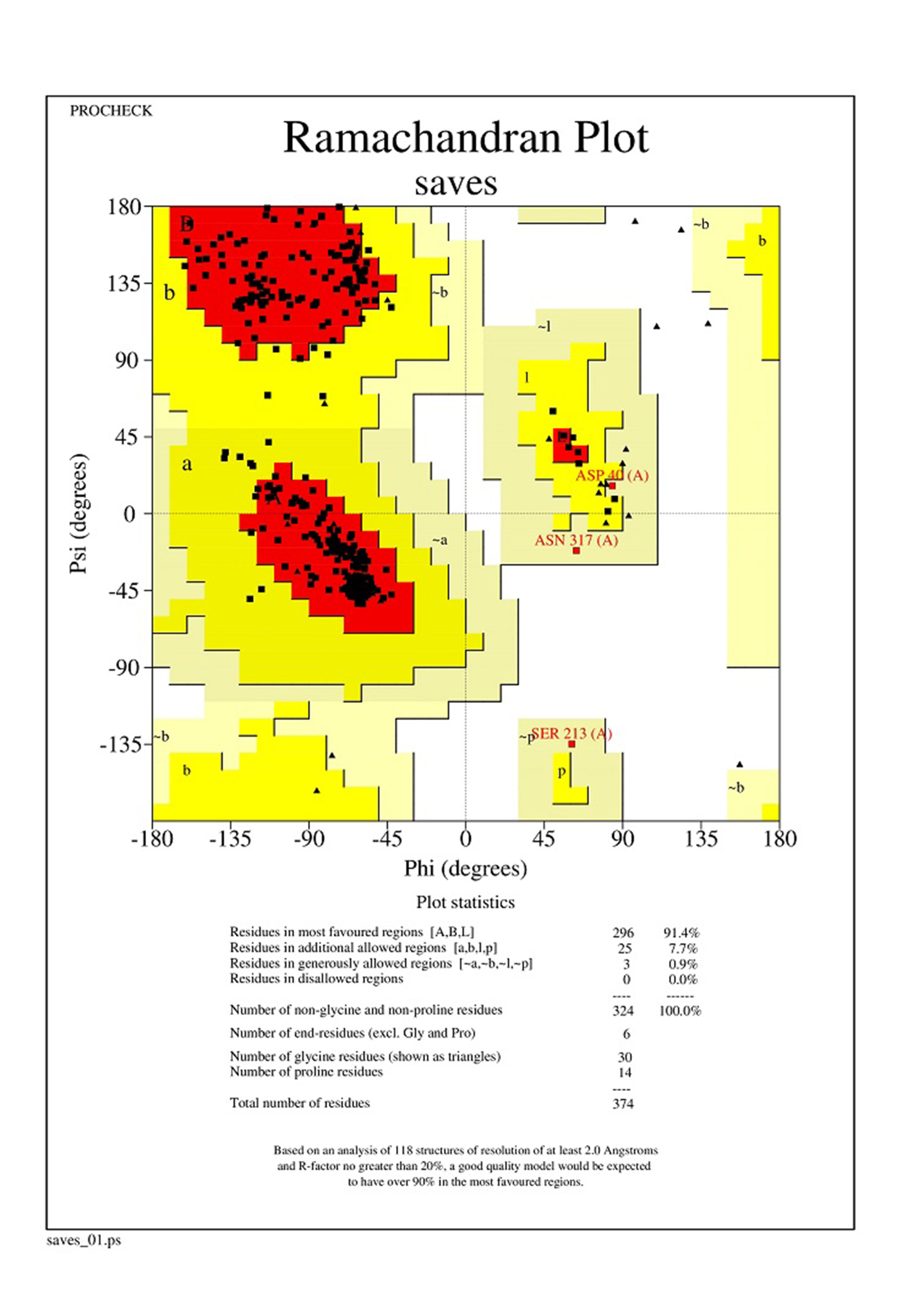

Supplement: Supplementary Figure 3 — Ramachandran plot of T. annulata HDAC1 homology model. [file Image_3.JPEG]
